# Expression of CXC chemokine receptor-4 and forkhead box 3 in neuroblastoma cells and response to chemotherapy

**DOI:** 10.3892/ol.2014.2028

**Published:** 2014-04-03

**Authors:** JING SUN, CHEN FENG, WEIWEI LIAO, HAO ZHANG, SUOQIN TANG

**Affiliations:** 1 Department of Pediatrics, Chinese PLA General Hospital 304, Beijing 100037, P.R. China; 2 Department of Pediatrics, Chinese PLA General Hospital, Beijing 100853, P.R. China

**Keywords:** CXC chemokine receptor-4, neuroblastoma, forkhead box 3, chemotherapy, gene expression

## Abstract

Current evidence indicates that the abnormal expression of chemokines or their receptors, such as CXC chemokine receptor-4 (CXCR4), is positively correlated with the development, progression and metastasis of tumor cells. However, the role of CXCR4 in neuroblastoma and its response to chemotherapy remain largely unclear. In addition, forkhead box 3 (Foxp3), a transcription factor associated with T cell tolerance, is expressed in tumor cells and plays a role in the immune evasion of cancers. The present study aimed to examine the expression of CXCR4 and Foxp3 in the LAN-5 and SK-N-SH neuroblastoma cell lines. The effects of chemotherapy drugs, cyclophosphamide (CTX) and pirarubicin (THP), on the expression of these two genes were also investigated. Our findings indicated that CXCR4 and Foxp3 were highly expressed in LAN-5 and SK-N-SH cells. Following treatment with CTX and THP, the protein expression of CXCR4 in LAN-5 and SK-N-SH cells was significantly decreased (P<0.05). The expression of Foxp3 in LAN-5 cells was also significantly downregulated by CTX and THP treatment (P<0.05). Therefore, the high expression of CXCR4 and Foxp3 in LAN-5 and SK-N-SH cells and their subsequent downregulation following administration of the chemotherapy agents suggests that the chemokine receptors, CXCR4 and Foxp3, may be involved in the metastasis and tumor evasion of neuroblastoma. Further studies should investigate the expression of CXCR4 and Foxp3 in patient samples.

## Introduction

Proinflammatory cytokines, including chemokines, attack inflammatory cells and regulate hematopoietic cell migration to bone marrow or lymph nodes and neuronal migration (1,2). Abnormal expression of chemokines or their receptors is positively correlated with the development, progression and metastasis of tumor cells (3,4). CXC chemokine receptor-4 (CXCR4) is highly expressed on the surface of several different types of tumors (5). CXCL12 (CXC motif chemokine 12) [also known as stromal cell-derived factor (SDF-1)] has been identified as the specific ligand of CXCR4 and it is likely that the CXCR4/CXCL12 axis is involved in the development, progression and metastasis of tumors (6,7). Forkhead box 3 (Foxp3) is a transcription factor that is required for the differentiation of regulatory T cells (Tregs) and is associated with T-cell tolerance and immune suppression (8). Emerging evidence indicates that Foxp3 is expressed in tumor cells and may play a role in the tumor evasion of cancers (9–11).

Neuroblastoma is one of the most common types of solid tumor found in children worldwide. As metastasis of neuroblastoma occurs at a high frequency (12), metastasis is the ultimate step in the progression of tumor cells toward autonomy from the host, it is required to identify the mechanisms underlying tumor cell metastasis. Although the abnormal expression of CXCR4 and Foxp3 may be involved in the metastasis and immune evasion of other types of tumors (13–16), their role in neuroblastoma and their response to chemotherapy remain largely unclear. Thus, the present study aimed to examine the expression of CXCR4 and Foxp3 in neuroblastoma cell lines LAN-5 and SK-N-SH. The effects of chemotherapy drugs, such as cyclophosphamide (CTX) and pirarubicin (THP), on the expression of CXCR4 and Foxp3 were also investigated.

## Materials and methods

### Cell lines and culture condition

The LAN-5 neuroblastoma cell line was kindly provided by Dr Stuart Elliott Siegel (Children’s Hospital Los Angeles, Los Angeles, CA, USA) and the origin has been described previously (17,18); the SK-N-SH cell line was purchased from American Type Culture Collection (Cambridge, MA, USA). The cells were maintained in RPMI-1640 (Gibco, Paisley, UK) supplemented with 10% fetal calf serum (FCS; Gibco) in an atmosphere of 5% CO_2_ at 37°C.

### Reagents

CTX was purchased from Shanxi Pude Pharmaceuticals Co., Ltd. (Shanxi, China) and THP was purchased from Shenzhen Wanle Pharmaceuticals Co., Ltd. (Shenzhen, China). The polyclonal mouse anti-human CXCR4 antibody was purchased from BioLegend (San Diego, CA, USA). TRIzol was purchased from Invitrogen Life Technologies (Carlsbad, CA, USA). The reverse transcription kit (ReverTra Ace-α) and SYBR Green Real-time PCR kit were purchased from Toyobo Co., Ltd. (Osaka, Japan).

### Quantitative polymerase chain reaction (qPCR)

A total of 5×10^6^ cells were collected. Total RNA was isolated using TRIzol reagent and transcribed into cDNA using a reverse transcription kit (Toyobo Co., Ltd.). qPCR was performed using 2× SYBR Green Real-time PCR Master Mix according to the manufacturer’s instruction (Toyobo, Co., Ltd). For amplification of CXCR4, the sense and antisense primers were 5′-CGTGCCCTCCTGCTGACTATT-3′ and 5′-GCCAACCATGATGTGCTGAA-3′, respectively. The forward and reverse primers for Foxp3 were 5′-GTTCACACGCATGTTTGCCTTC-3′ and 5′-GCACAAAGCACTTGTGCAGACTC-3′, respectively. The forward and reverse primers for the control gene, human GAPDH, were 5′-AATGGAAATCCCATCACCATCT-3′ and 5′-CGCCCCACTTGATTTTGG-3′, respectively. PCR was performed in a MX3000 machine (Eppendorf, Hamburg, Germany) with the following conditions: 40 cycles of 95°C for 30 sec, 55°C for 30 sec and 72°C for 30 sec. Each sample was determined in triplicate. Target mRNA expression was calculated as target gene copies/GAPDH copies. The relative standard curve method was used to determine the relative mRNA expression of CXCR4 and Foxp3 genes.

### Cell proliferation assays

The LAN-5 or SK-N-SH cells in RPMI-1640 medium supplemented with 10% FCS were plated at 1×10^4^ cells/well in a 96-well flat-bottom tissue culture plate and incubated for 24 h at 37°C. CTX or THP was added to the culture medium and the cells were incubated for 72 h at 37°C. To each well, 10 μl of 5 mg/ml MTT (Sigma-Aldrich, St. Louis, MO, USA) was added and, after 6 h of incubation, 100 μl of dimethylsulfoxide (Sigma-Aldrich) was added to each well and absorbance was measured at 570 nm on a microplate reader (Bio-Rad Laboratories, Inc., Hercules, CA, USA).

### Flow cytometry

A total of 1×10^6^ LAN-5 or SK-N-SH cells were collected and 1 μl of mouse phycoerythrin (PE) labeled monoclonal anti-human CXCR4 antibody (BioLegend) was added to each sample and incubated at 4°C for 30 min. The cells were washed three times and analyzed on a Becton-Dickinson FACSCalibur flow cytometer (BD Biosciences, Franklin Lakes, NJ, USA).

To detect the intracellular Foxp3 expression, the cells were first permeablized with the permeabilization buffer according to the manufacturer’s instructions (eBioscience, San Diego, CA, USA). Mouse PE labeled monoclonal anti-human Foxp3 antibody (1 μl; BioLegend) was added to the cells and incubated at 4°C for 30 min. After three washes, the cells were analyzed on the FACSCalibur flow cytometer.

### Statistical analysis

Differences between cases and controls regarding the means and proportions were compared using Student’s t-test and the χ^2^ test. SPSS software, version 17.0 (SPSS, Inc., Chicago, IL, USA) was used for statistical analysis. P<0.05 was considered to indicate a statistically significant difference.

## Results

### Expression of CXCR4 and Foxp3 in LAN-5 and SK-N-SH cells

**Figure 1:**
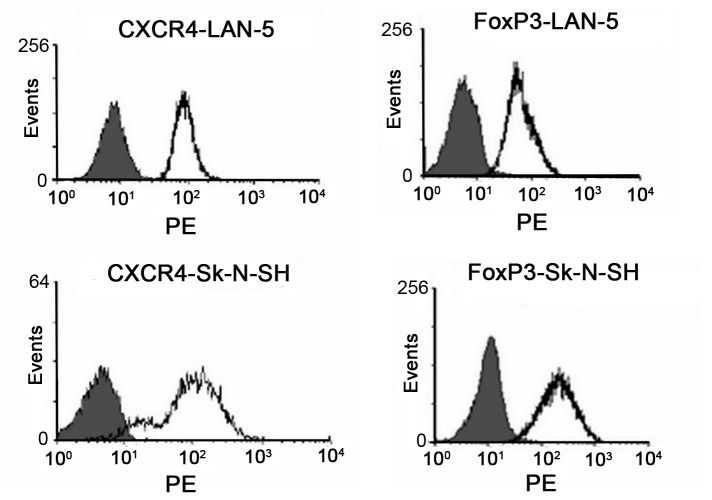
CXCR4 and Foxp3 expression in the LAN-5 and SK-N-SH neuroblastoma cell lines detected by fluorescence-activated cell sorting. CXCR4, CXC chemokine receptor-4; Foxp3, forkhead box 3; PE, R-Phycoerythrin.

To investigate the role of CXCR4 in the progression and metastasis of neuroblastoma, the expression of CXCR4 in LAN-5 and SK-N-SH cells was analyzed by FACS. As shown in Fig. 1, CXCR4 was highly expressed in the two cell lines. Foxp3 was also highly expressed in the LAN-5 and SK-N-SH cell lines (Fig. 1).

### Chemotherapeutic drugs inhibit the proliferation of neuroblastoma cells

The inhibition effects of CTX and THP on the proliferation of LAN-5 and SK-N-SH cells were analyzed by the MTT assay. CTX inhibited the proliferation of LAN-5 and SK-N-SH cells at the half maximal inhibitory (IC_50_) value of 6.7 and 3.8 μmol/l, respectively. THP inhibited the proliferation of LAN-5 and SK-N-SH cells at the IC_50_ value of 0.067 μg/ml.

### Chemotherapy treatment downregulates the expression of CXCR4 and Foxp3

**Figure 2:**
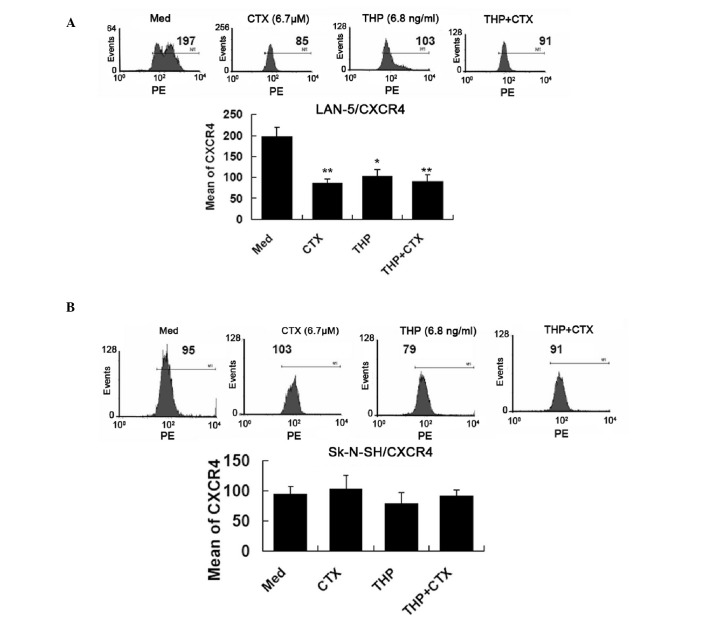
CTX and THP downregulated the protein expression of CXCR4 detected by fluorescence-activated cell sorting. ^*^P<0.05 and ^**^P<0.01, compared with the control. CTX, cyclophosphamide; THP, pirarubicin; Med, medium control.

**Figure 3:**
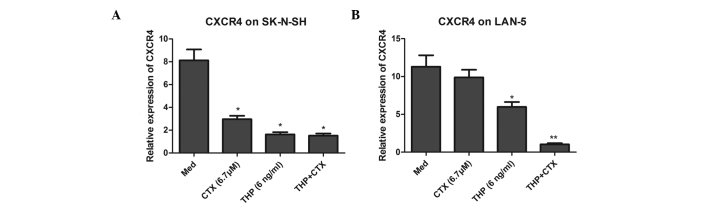
CTX and THP downregulated the mRNA expression of CXCR4 detected by quantitative polymerase chain reaction. .^*^P<0.05 and ^**^P<0.01, compared with the control. CTX, cyclophosphamide; THP, pirarubicin; CXCR4, CXC chemokine receptor-4; Med, medium control.

As shown in Fig. 2, at the protein level, CTX and THP decreased the expression of CXCR4 on the surface of LAN-5 cells (P<0.05). However, CTX and THP did not affect the protein level of CXCR4 in the SK-N-SH cells. THP, but not CTX, significantly reduced the mRNA level of CXCR4 in LAN-5 cells. However, THP and CTX significantly downregulated the mRNA expression of CXCR4 in the SK-N-SH cells (Fig. 3).

**Figure 4:**
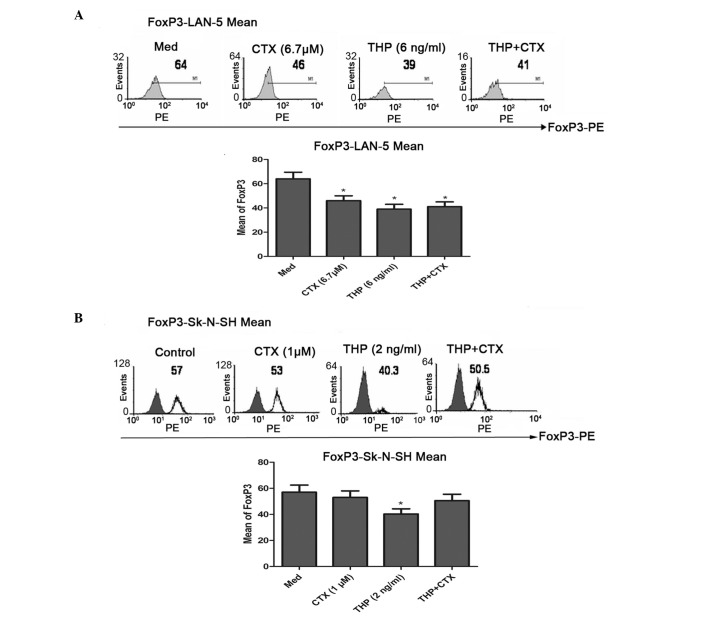
CTX and THP downregulated the protein expression of Foxp3 detected by fluorescence-activated cell sorting. ^*^P<0.05 and ^**^P<0.01, compared with the control. Med, medium control; CTX, cyclophosphamide; THP, pirarubicin; Foxp3, forkhead box 3; PE, R-Phycoerythrin.

**Figure 5:**
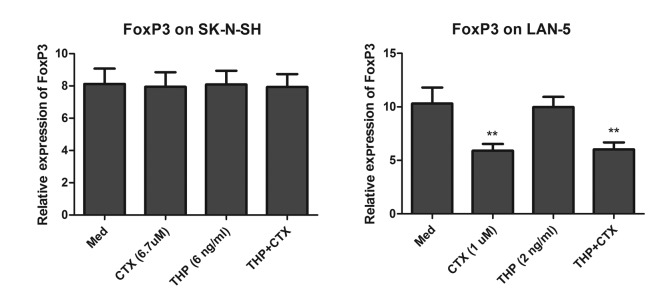
CTX and THP downregulated the mRNA expression of Foxp3 detected by quantitative polymerase chain reaction. ^*^P<0.05 and ^**^P<0.01, compared with the control. Med, medium control; CTX, cyclophosphamide; THP, pirarubicin; Foxp3, forkhead box 3.

FACS data showed that CTX and THP significantly downregulated the expression of Foxp3 in the LAN-5 cells (P<0.05) (Fig. 4), while CTX and THP did not affect the expression of Foxp3 in the SK-N-SH cells. qPCR analysis showed that only CTX significantly downregulated the expression of Foxp3 in the LAN-5 cells (Fig. 5).

## Discussion

Neuroblastoma is a type of cancer that originates in certain primitive nerve cells found in an embryo or fetus. This type of cancer occurs in infants and young children. It is rarely found in children >10 years old (19,20). Although more than one half of children have localized tumors with a good prognosis, the remaining patients have metastatic disease with a poor long-term survival rate of ~30% worldwide (21,22). Therefore the presence of metastatic diseases in children with neuroblastoma is associated with a poor prognosis. The most frequent distant metastatic sites are bone marrow and cortical bone (23). However, the mechanisms underlying the high frequency of metastasis in patients with neuroblastoma remain unclear. Understanding the mechanisms of bone and bone marrow metastasis of neuroblastoma may affect current and future therapies of this disease.

CXCR4, a receptor for a chemokine CXCL12, is important for the migration of hematopoietic cells to bone marrow and neuronal migration (24,25). Emerging evidence suggests that CXCR4 may also play an important role in the metastasis of a variety of tumors. CXCR4 expression has been found to be associated with bone marrow metastases of breast (26) and prostate (27) cancer, and rhabdomyosarcoma (28). Thus, studying the role of CXCR4 in the metastasis of neuroblastoma is of great importance. In the present study, CXCR4 was found to be highly expressed in the LAN-5 and SK-N-SH neuroblastoma cell lines. Notably, the expression of CXCR4 was decreased after the cells were treated with chemotherapy drugs, CTX and THP, in association with the inhibition of cell proliferation; therefore, the expression of CXCR4 may be involved in the metastasis of neuroblastoma.

Immune evasion of tumors contributes to the survival of cancer cells. Tregs plays an important role in the suppression of immune responses and are also involved in immune evasion in patients with cancer. The increased prevalence of Tregs may be induced in patients with tumors (29). The transcription factor, Foxp3, is a key factor for the differentiation of Tregs and is expressed in T cells only. However, previous studies have demonstrated that Foxp3 was also expressed in tumor cells, such as pancreatic cancer (30), melanoma (31) and other tumor cell lines (32,33). The Foxp3-expressing cancer cells inhibited the proliferation of CD4^+^CD25^−^ T cells, potentially contributing to immune evasion of the tumor cells. However, the role of Foxp3 in neuroblastoma is largely unknown. In this study, the expression of Foxp3 in neuroblastoma cells was investigated and our findings indicated that Foxp3 was expressed in the LAN-5 and SK-N-SH neuroblastoma cell lines. When the proliferation of tumor cells was inhibited by CTX and THP, the expression of Foxp3 also significantly decreased. Merlo *et al* reported that Foxp3 expression may be associated with the metastatic potential of the tumor rather than suppression of a specific immune response (34). The authors proposed that Foxp3 expressed in cancer cells may modulate expression of chemokine receptors and other genes, and thus influence invasion and metastasis of tumor cells. In our study, Foxp3 and CXCR4 were expressed in neuroblastoma cells; therefore, it may be possible that Foxp3 expressed in these cells upregulated CXCR4 and contributed to the higher frequency of neuroblastoma.

In conclusion, the present study demonstrates that CXCR4 and Foxp3 exhibit higher expression in neuroblastoma cells, and therefore, exposure to chemotherapy agents may reduce their expression. In addition, the results suggest that CXCR4 and Foxp3 may present as potential targets for neuroblastoma chemotherapy.
